# DNA barcoding and the differentiation between North American and West European *Phormia regina* (Diptera, Calliphoridae, Chrysomyinae)

**DOI:** 10.3897/zookeys.365.6202

**Published:** 2013-12-30

**Authors:** Kurt Jordaens, Gontran Sonet, Yves Braet, Marc De Meyer, Thierry Backeljau, Frankie Goovaerts, Luc Bourguignon, Stijn Desmyter

**Affiliations:** 1 Royal Museum for Central Africa, Department of Biology (JEMU), Leuvensesteenweg 13, 3080 Tervuren, Belgium; 2 University of Antwerp, Evolutionary Ecology Group, Groenenborgerlaan 171, 2020 Antwerp, Belgium; 3 Royal Belgian Institute of Natural Sciences, OD Taxonomy and Phylogeny (JEMU), Vautierstraat 29, 1000 Brussels, Belgium; 4 National Institute of Criminalistics and Criminology, Vilvoordsesteenweg 100, 1120 Brussels, Belgium

**Keywords:** Black fly, COI, COII, cyt *b*, 16S, 28S, ITS2

## Abstract

*Phormia regina* (the black fly) is a common Holarctic blow fly species which serves as a primary indicator taxon to estimate minimal post mortem intervals. It is also a major research model in physiological and neurological studies on insect feeding. Previous studies have shown a sequence divergence of up to 4.3% in the mitochondrial COI gene between W European and N American *P. regina* populations. Here, we DNA barcoded *P. regina* specimens from six N American and 17 W European populations and confirmed a mean sequence divergence of ca. 4% between the populations of the two continents, while sequence divergence within each continent was a ten-fold lower. Comparable mean mtDNA sequence divergences were observed for COII (3.7%) and cyt *b* (5.3%), but mean divergence was lower for 16S (0.4–0.6%). Intercontinental divergence at nuclear DNA was very low (≤ 0.1% for both 28S and ITS2), and we did not detect any morphological differentiation between N American and W European specimens. Therefore, we consider the strong differentiation at COI, COII and cyt *b* as intraspecific mtDNA sequence divergence that should be taken into account when using *P. regina* in forensic casework or experimental research.

## Introduction

Forensic entomology uses the larval and pupal developmental stages of insects sampled on a corpse to estimate a minimum post-mortem interval (PMImin) of the corpse (Amendt et al. 2004, 2007). This requires i) detailed and accurate knowledge of the developmental rate of the species of forensic interest under different temperature conditions (Charabidze 2012), and ii) identification tools by which the different immature insect stadia can be identified (Catts 1992). Blowflies (family Calliphoridae) are among the most common insects found on dead bodies shortly after death. The species differ in their developmental times and have therefore a high potential for the accurate estimation of the PMImin. Unfortunately, several forensically important blow fly species can hardly be distinguished morphologically, especially in the larval and pupal stages (e.g. Catts 1992). To improve the success and reliability of identifications, a number of molecular techniques and tools have been explored to identify forensically important species (Wells and Stevens 2008, reviewed in Jordaens et al. in press).

Currently, the most popular molecular method for organismal identification is DNA barcoding, which was promoted by Hebert et al. (2003a, b) as a standardized molecular identification tool for all animals. It refers to establishing species-level identifications by sequencing a fragment of the mitochondrial cytochrome *c* oxidase subunit I (COI) gene, the “DNA barcode”, into a taxonomically unknown specimen and performing comparisons with a reference library of barcodes of well-identified species. COI barcodes (and other fragments of COI) indeed have been successfully applied in the identification of many calliphorid species (e.g. Wallman and Donnellan 2001, Wells and Sperling 2001, Nelson et al. 2007, Wells and Williams 2007, Harvey et al. 2008, Desmyter and Gosselin 2009, DeBry et al. 2013). Yet, COI fails to unambiguously discriminate among several calliphorid species pairs (e.g. Nelson et al. 2007, see also the Discussion) and the use of alternative identification tools (e.g. other genes) could be necessary to acquire correct identifications.

The monophyly of Calliphoridae has been questioned for many years (e.g. Griffiths 1982) and paraphyly or polyphyly was suggested by a morphology-based parsimony analysis (Rognes 1997). Nonmonophyly was also found in a molecular phylogenetic analysis of the Calyptratae with Calliphoridae being polyphyletic with respect to the Tachinidae and Rhinophoridae. Within this ‘calliphorid-tachinid-rhinophorid’ clade, the subfamily Chrysomyinae was para- or polyphyletic (Kutty et al. 2010). The Chrysomyinae comprises two tribes, Chrysomyini and Phormiini, of which the Phormiini has three genera (Table 1). *Phormia regina* (Meigen, 1826) (black fly) is the only species in the monotypic genus *Phormia*. It is a Holarctic blow fly species that is commonly found on human or animal faeces (Coffey 1966) and that is frequently found on corpses. It therefore serves as a primary species to estimate the PMImin (e.g. Byrd and Allen 2001). Further, the species also plays an important role in secondary myasis in cattle (e.g. Francesconi and Lupi 2012) and is used in maggot therapy (Knipling and Rainwater 1937).

**Table 1.** Taxonomy of the subfamily Chrysomyinae (family Calliphoridae) with indication of the number of DNA sequences (the number of haplotypes is given in parentheses) for each of the species used in this study (numbers combined from this study and GenBank) and for each of the gene fragments studied. No. ind. = number of individuals; No. hapl. = number of haplotypes; No. spp. = number of species.

	Genus/species	COI	COII	16S		cyt *b*	ITS2	28S
				251 bp	350 bp			
Chrysomyini	*Chloroprocta* Wulp, 1896							
	*Chloroprocta idioidea* (Robineau-Desvoidy, 1830)	2(2)					1(1)	1(1)
	*Chrysomya* Robineau-Desvoidy, 1830							
	*Chrysomya albiceps* (Wiedemann, 1819)	3(2)	1(1)				2(1)	2(1)
	*Chrysomya bezziana* Villeneuve, 1914	5(2)	1(1)			10(6)	2(1)	2(2)
	*Chrysomya cabrerai* Kurahashi & Salazar, 1977	1(1)						
	*Chrysomya chani* Kurahashi, 1979	1(1)					11(2)	
	*Chrysomya chloropyga* (Wiedemann, 1818)	1(1)						2(2)
	*Chrysomya defixa* (Walker, 1856)	1(1)						
	*Chrysomya flavifrons* (Aldrich, 1925)	3(2)	1(1)				4(2)	
	*Chrysomya greenbergi* Wells & Kurahashi, 1996	1(1)						
	*Chrysomya incisularis* (Macquart, 1851)	9(2)	2(2)				1(1)	
	*Chrysomya latifrons* (Malloch, 1927)	6(2)	1(1)				5(1)	
	*Chrysomya megacephala* (Fabricius, 1794)	79(11)	28(7)	66(31)	20(3)	2(2)	42(3)	4(2)
	*Chrysomya nigripes* Aubertin, 1932	9(7)	3(3)				7(1)	
	*Chrysomya norrisi* James, 1971	1(1)	1(1)					
	*Chrysomya pacifica* Kurahashi, 1991	1(1)					1(1)	
	*Chrysomya pinguis* (Walker, 1858)	7(4)	1(1)				14(2)	
	*Chrysomya putoria* (Wiedemann, 1830)	2(2)	1(1)			1(1)	2(1)	
	*Chrysomya rufifacies* (Macquart, 1843)	25(10)	45(9)	10(5)	1(1)		14(1)	2(2)
	*Chrysomya saffranea* (Bigot, 1877)	7(2)	1(1)				8(2)	
	*Chrysomya semimetallica* (Malloch, 1927)	11(5)	3(2)				10(2)	
	*Chrysomya thanomthini* Kurahashi & Tumrasvin, 1977	1(1)						
	*Chrysomya varipes* (Macquart, 1851)	7(6)	6(2)				1(1)	
	*Chrysomya villeneuvi* Patton, 1922						7(1)	
	*Cochliomyia* Townsend, 1915							
	*Cochliomyia hominivorax* (Coquerel, 1858)	78(73)	65(62)			2(1)	90(24)	2(1)
	*Cochliomyia macellaria* (Fabricius, 1775)	3(3)	1(1)				1(1)	4(1)
	*Compsomyiops* Townsend, 1918							
	*Compsomyiops calipes* (Bigot, 1877)	1(1)	1(1)					
	*Compsomyiops fulvicrura* (Robineau-Desvoidy, 1830)			1(1)	1(1)			1(1)
	*Hemilucilia* Brauer, 1895							
	*Hemilucilia segmentaria* (Fabricius, 1805)	1(1)					1(1)	1(1)
	*Hemilucilia semidiaphana* (Rondani, 1850)	1(1)					1(1)	1(1)
	*Paralucilia* Brauer & Bergenstamm, 1891							
	*Paralucilia paraensis* (Mello, 1969)	1(1)						
	*Trypocalliphora* Peus, 1960							
	*Trypocalliphora braueri* (Hendel, 1901)	1(1)						
Phormiini	*Phormia* Robineau-Desvoidy, 1830							
	*Phormia regina* (Meigen, 1826)	48(20)	30(9)	15(2)	15(2)	17(10)	36(2)	38(2)
	*Protophormia* Townsend, 1908							
	*Protophormia terraenovae* (Robineau-Desvoidy, 1830)	17(7)	1(1)	2(2)	1(1)		1(1)	4(2)
	*Protocalliphora* Hough, 1899							
	*Protocalliphora azurea* (Fallen, 1817)		2(2)		1(1)	1(1)		1(1)
	*Protocalliphora occidentalis* Whitworth, 2003		1(1)					
	*Protocalliphora sialia* Shannon & Dobroscky, 1924		1(1)	1(1)				
	*Protocalliphora* sp.			1(1)				
Total no. ind.		339	194	95	39	32	263	66
Total no. hapl.		180	108	42	9	20	55	21
Total no. spp.		36		20	6	6	5	24

*Phormia regina* is a highly mobile species that is abundant in North American areas with cool spring and fall temperatures and in warmer areas, but then at higher altitudes (Hall 1948, Brundage et al. 2011). The developmental time of *Phormia regina* seems highly variable and could be influenced by a number of environmental variables (Kamal 1958, Greenberg 1991, Anderson 2000, Byrd and Allen 2001, Nabity et al. 2007, Núñez-Vázquez et al. 2013). Using amplified fragment length polymorphisms (AFLP), Picard and Wells (2009) studied the population genetic structure of N American *Phormia regina* and found that the N American populations were panmictic but with significant temporal genetic differences within populations, even over short periods of time. They therefore suggested that part of the variation in developmental times and growth curves that was observed in laboratory studies is not only due to local environmental (i.e. laboratory) conditions, but also to differences in the genetic composition of the laboratory stocks. This finding is important for forensic sciences since it shows that forensically relevant ecological data from one population (i.e. from a forensic case) cannot be extrapolated to other populations (i.e. to other forensic cases). Interestingly, Desmyter and Gosselin (2009) found a 4.2% sequence divergence at a 304 bp COI fragment between N American and W European specimens. Subsequently, Boehme et al. (2012) found a similar sequence divergence (range: 3.5%–4.31%) at the COI barcodes between N American and W European *Phormia regina* specimens.

Because high COI sequence divergences are often indicating species level differentiation (e.g. Hebert et al. 2003a, b), the strong COI differentiation between N American and W European *Phormia regina* specimens calls for a taxonomic re-assessment. We therefore studied DNA sequence variation in mitochondrial and nuclear DNA, and examined morphological differentiation between N American and W European populations of *Phormia regina* to i) provide additional DNA barcodes for *Phormia regina*, ii) examine molecular differentiation between N American and W European specimens in other genes, and iii) assess whether the COI differentiation is correlated with morphological differentiation. The taxonomy of *Phormia regina* is then re-evaluated in the light of these results.

## Material and methods

### Specimen collection and morphological examination

**Figure 1.**
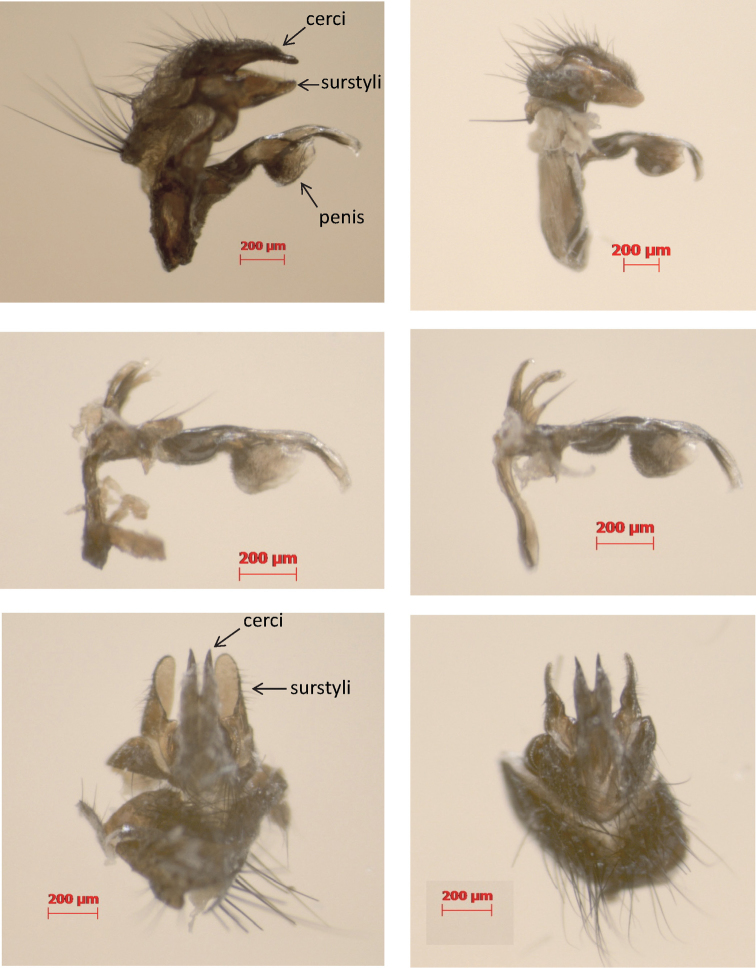
Lateral (top) and dorsal (bottom) view of the male copulatory organs of *Phormia regina* from W Europe (left) and N America (right) with a detail of the penis (middle).

Sixty-one adult individuals of *Phormia regina* were captured at several localities in N America (Indiana, Texas, Virginia, Washington, Wyoming) and W Europe (Belgium, France, Germany) and stored in > 70% ethanol (Appendix 1 – Supplementary table 1). The individuals were qualitatively scored for the color of 11 external characters (Table 2). In addition, we dissected the male copulatory organs of five W European and five N American individuals to study the general shape of the penis, cerci and surstyli (Figure 1).

**Table 2.** Color scoring of eleven external morphological characters of adult W European and N American *Phormia regina*.

Character	W Europe and N America
calypters	white
first spiraculum	white to yellow
thoracic dorsum	metallic green-bluish to dark green
scutellum	dark green
legs	black
abdomen	metallic green-bluish
facial ridge	red-brown
gena	black
postgena	black
first antennal segment	dark-brown to black
second antennal segment	white-grey

### DNA sequence analysis

DNA was extracted from on one or two legs. The remaining parts of the vouchers are kept at the NICC (National Institute of Criminalistics and Criminology – Brussels, Belgium) as pinned material. Genomic DNA was extracted using the NucleoSpin Tissue kit (Macherey-Nagel). A fragment of 721 bp from the 5’-end of the COI gene, including the standard barcode region (Hebert et al. 2003a, b), was amplified using primer pair TY-J-1460 and C1-N-2191 (Sperling et al. 1994, Wells and Sperling 2001). Five other DNA markers were sequenced for a more limited set of samples (Appendix 1 – Supplementary table 1). Fragments of the mitochondrial 16S ribosomal RNA (16S), cytochrome *c* oxidase subunit II (COII), and cytochrome *b* (cyt *b*) genes, and of the nuclear ribosomal internal transcribed spacer 2 (ITS2) and fragment D1–D2 of the 28S ribosomal RNA (28S) were amplified using primer pairs 16Sf.dip/16Sr.dip (Kutty et al. 2007), C2-J-3138/TK-N-3775 (Wells and Sperling 2001), CB1-SE/PDR-WR04 (Ready et al. 2009), ITS2F.dip/ITS2R (Song et al. 2008) and D1F/D2R (Stevens and Wall 2001), respectively.

Each 25 µl PCR reaction was prepared using 1 × PCR buffer, 0.2 mM dNTPs, 0.4 μM of each primer, 2.0 mM MgCl_2_, 0.5 U of Taq DNA polymerase (Platinum®, Invitrogen), 2–4 µl DNA template (DNA was stored in 100 µl of elution buffer) and enough mQ-H_2_O to complete the total PCR reaction volume. The thermal cycler program consisted of an initial denaturation step of 4 min at 94 °C, followed by 30–40 cycles of 45–60 s at 94 °C, 30–60 s at a fragment depending annealing temperature and 90 s at 72 °C; with a final extension of 7 min at 72 °C. The annealing temperatures were 45 °C for COI and COII, 48 °C for 16S and cyt *b*, 50 °C for ITS-2 and 55 °C for 28S. PCR products were cleaned using the NucleoFast96 PCR® kit (Macherey-Nagel) and bidirectionally sequenced on an ABI 3130 Genetic Analyzer (Applied Biosystems) using the BigDye® Terminator Cycle Sequencing Kit v3.1. Together with the *Phormia regina* specimens we also collected several *Protophormia terraenovae* specimens that were also sequenced to increase the number of material for comparison (Appendix 1 – Supplementary table 1). Sequences were assembled in SeqScape v2.5 (Applied Biosystems) and deposited in GenBank under accession numbers KF908069–KF908124 (COI), KF908126–KF908152 (COII), KF908153–KF908169 (cyt *b*), KF908054–KF908068 (16S), KF908170–KF908203 (ITS2), and KF908204–KF908237 (28S).

Phormiini and its sister clade Chrysomyini form the Chrysomyinae (Singh and Wells 2011a, b). We therefore downloaded from GenBank (and for all genes) all available sequences (at 11 July 2013) of the Phormiini (genera *Phormia*, *Protophormia* and *Protocalliphora*) and of the Chrysomyini (genera *Chloroprocta*, *Chrysomya*, *Cochliomyia*, *Compsomyiops*, *Hemilucilia*, *Paralucilia* and *Trypocalliphora*) to allow comparison with closely related taxa (Table 1). Sequences were aligned in MAFFT v7 (Katoh and Standley 2013). Sequences with > 5 ambiguous positions were discarded and each dataset was trimmed to equal sequence length (Table 3). The 16S dataset was trimmed at 251 bp and at 350 bp to yield a higher number of Chrysomyinae haplotypes for the latter dataset (i.e. 22 vs. 42 unique haplotypes; six species in the ingroup for both datasets). Alignments are available as fasta files in the online Appendix 2 text file. Unique sequences (haplotypes) were selected in DAMBE5 (Xia 2013). Nucleotide sequence divergences within and between species (based on the haplotypes) were calculated using the uncorrected p-distances in MEGA v5.05 (Tamura et al. 2011). For these calculations we excluded haplotypes that were not identified to the species level (one *Protocalliphora* sp. for COI) or that were most likely identification errors (for details see the Results). MEGA v5.05 was also used to construct Neighbour-Joining (NJ) trees (Saitou and Nei 1987) using the p-distances with complete deletion of positions with ambiguities and alignment gaps (indels). Relative branch support was evaluated with 1000 bootstrap replicates (Felsenstein 1985). In all analyses, several *Lucilia* spp. or *Calliphora* spp. sequences from GenBank were added as outgroups, and for COI we also used *Lucilia sericata* NICC0390 as outgroup (GenBank accession number KF908125). Author names of all species are provided in Table 1.

**Table 3.** Description of the *Phormia regina* and other Chrysomyinae DNA sequences (including those retrieved from GenBank) for each of the gene fragments.

Marker	COI	COII	16S		cyt *b*	ITS2 (without indels)	28S (without indels)
			251 bp	350 bp			
Fragment size (bp)	655	472	251	350	512	380 (224)	633 (592)
*Phormia regina*							
Total							
No of sequences	50	30	15	15	17	36	37
No of haplotypes	20	9	2	4	10	4	2
**North America (NA)**							
No of sequences	27	27	11	11	10	25	23
No of haplotypes	14	7	1	3	7	1	2
Mean intra-NA distances (%)	0.004	0.004	-	0.004	0.005	-	0.002
SE	0.001	0.002	-	0.003	0.002	-	0.002
min. – max.	0.002–0.008	0.002–0.006	-	0.003–0.006	0.002–0.008	-	0.002
**Europe (EU)**							
No of sequences	23	3	4	4	7	11	14
No of haplotypes	6	2	1	1	3	4(2)	1
Mean intra-EU distances (%)	0.003	0.002	-	-	0.002	0.002	-
SE	0.001	0.002	-	-	0.007	0.002	-
min. – max.	0.002–0.008	0.002	-	-	0.002–0.010	0.002	-
Mean p-distance between NA and EU	0.04	0.037	0.004	0.006	0.053	0.001	0.001
SE	0.007	0.008	-	0.003	0.009	0.001	0.001
min. – max.	0.036–0.044	0.034–0.042	0.004	0.005–0.009	0.047–0.061	0–0.004	0–0.002
Other Chrysomyinae							
Mean intraspecific p-distance	0.005	0.014	0.028	0.014	0.003	0.008	0.003
SE	0.009	0.014	0.009	-	0.002	0.005	0.004
min. – max.	0–0.042	0–0.037	0.018–0.036	0.014	0.002–0.005	0.004–0.015	0–0.010
Mean interspecific p-distance	0.066	0.046	0.038	0.023	0.079	0.085	0.007
SE	0.005	0.005	0.006	0.004	0.007	0.011	0.002
min. – max.	0.011–0.113	0.002–0.135	0.03–0.075	0.023–0.057	0.073–0.141	0.009–0.166	0–0.015

## Results

### Morphology

We did not detect morphological differences between N American and W European *Phormia regina* specimens in the 11 external color characters that we scored (Table 2). Also the male copulatory organs of W European and N American *Phormia regina* specimens were indistinguishable (Figure 1).

### DNA sequence analysis

**Figure 2.**
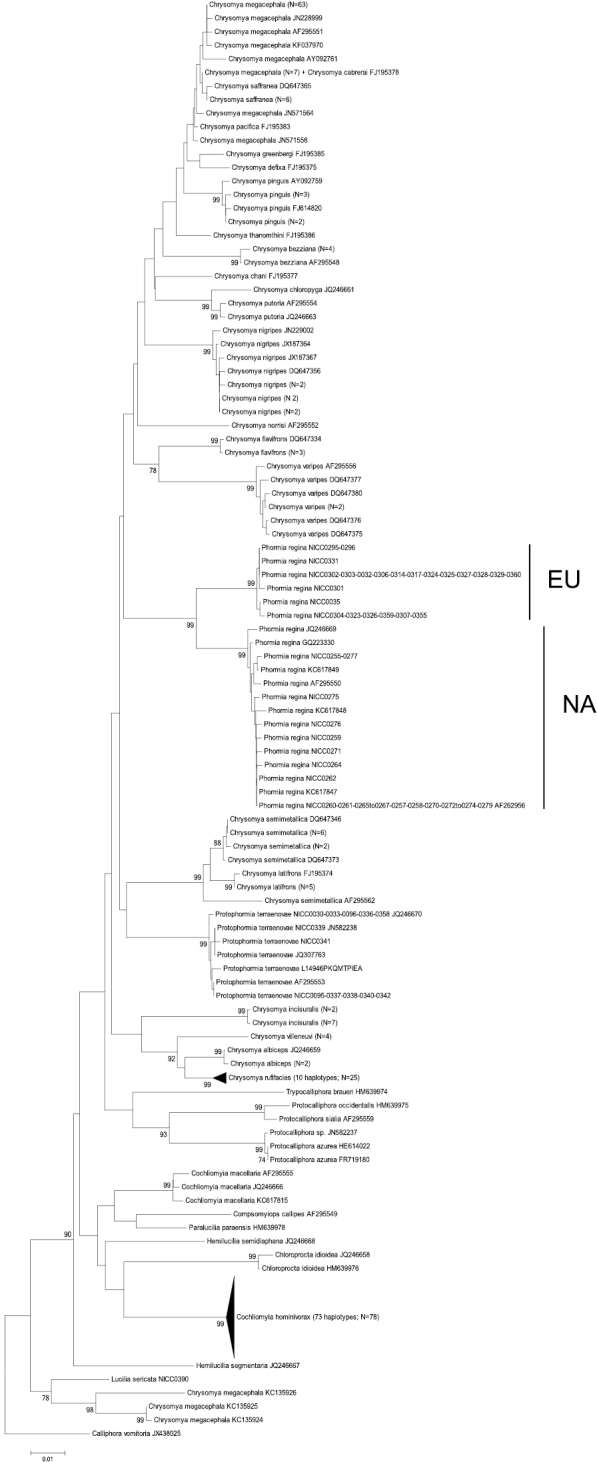
Neighbour-Joining tree (p-distances) of a 655 bp fragment of the mitochondrial cytochrome *c* oxidase subunit I (COI) gene. Bootstrap values ≥ 70% are shown at the nodes. N gives the number of specimens of that haplotype. EU = *Phormia regina* haplotypes from W Europe; NA = *Phormia regina* haplotypes from N America.

**Figure 3.**
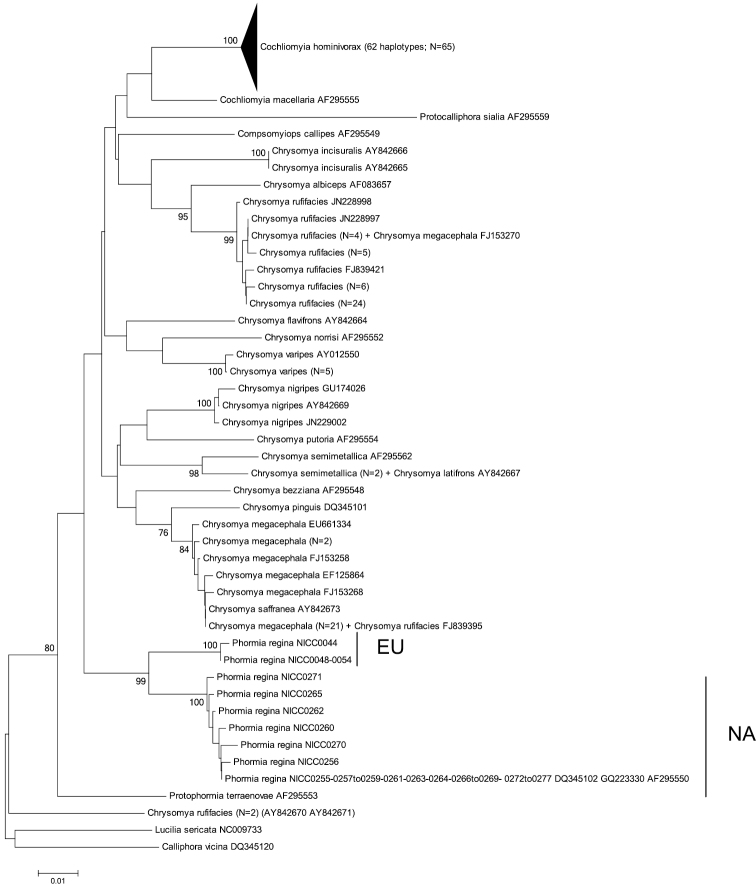
Neighbour-Joining tree (p-distances) of a 472 bp fragment of the mitochondrial cytochrome *c* oxidase subunit II (COII) gene. Bootstrap values ≥ 70% are shown at the nodes. N gives the number of specimens of that haplotype. EU = *Phormia regina* haplotypes from W Europe; NA = *Phormia regina* haplotypes from N America.

**Figure 4.**
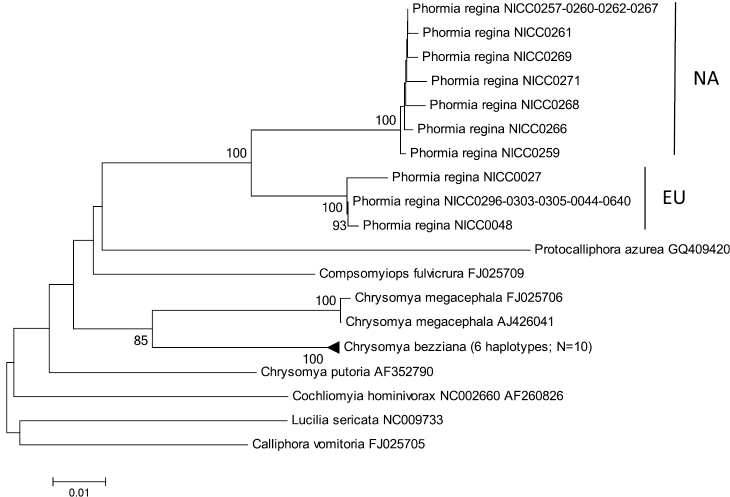
Neighbour-Joining tree (p-distances) of a 512 bp fragment of the mitochondrial cytochrome *b* (cyt *b*) gene. Bootstrap values ≥ 70% are shown at the nodes. N gives the number of specimens of that haplotype. EU = *Phormia regina* haplotypes from W Europe; NA = *Phormia regina* haplotypes from N America.

Basic information of the different datasets can be found in Table 3. There was only high bootstrap support for the monophyly of Chrysomyinae, Phormiini or Chrysomyini with 28S and a sister group relationship of *Phormia regina* and *Protophormia terraenovae* with ITS2. Yet, for all fragments, except for 28S, there was high bootstrap support for the monophyly of *Phormia regina* (Figures 2–4 and Appendix 1 – Supplementary figures 1–3).

**COI:** The COI NJ-tree showed two supported clades within *Phormia regina* (Figure 2). One clade (EU = Europe) comprised six haplotypes from Europe (23 specimens sequenced), while the other clade (NA = North America) comprised 14 haplotypes from N America (27 specimens sequenced). The seven NA haplotypes available in GenBank clustered within the NA clade. The mean p-distance between the EU and NA *Phormia regina* haplotypes was 0.04 ± 0.007 (Table 3). Sequence divergence in *Phormia regina* within each continent was approximately a ten-fold lower, viz. EU: 0.003 ± 0.001 – NA: 0.004 ± 0.001.

The mean p-distances between Chrysomyinae species pairs were: between three *Protocalliphora* spp.: 0.05 ± 0.006, 23 *Chrysomya* taxa: 0.06 ± 0.005 (the three *Chrysomya megacephala* specimens with GenBank accession numbers KC135924, KC135925 and KC135926 were treated as a different taxon from the other *Chrysomya megacephala* specimens because of a strong sequences divergence, viz. mean p-distance = 0.089 ± 0.01; see Figure 2), *Cochliomyia macellaria* – *Cochliomyia hominivorax*: 0.068 ± 0.009, and *Hemilucilia semidiaphana* – *Hemilucilia segmentaria*: 0.078 ± 0.001. The mean intra- and interspecific p-distances between all Chrysomyinae species (excluding *Phormia regina*) were 0.005 ± 0.009 and 0.066 ± 0.005, respectively (Table 3).

**COII**: The two EU and seven NA haplotypes of *Phormia regina* (from 30 specimens) formed two strongly supported clades (Figure 3) separated by mean p-distance of 0.037 ± 0.008 (Table 3). The three COII sequences from GenBank (from NA specimens) had the same haplotype as our NA specimens. Sequence divergence in *Phormia regina* within each continent was approximately a ten-fold lower, viz. EU: 0.002 ± 0.002 – NA: 0.004 ± 0.002 (Table 3). The mean p-distance between the 14 *Chrysomya* taxa was 0.059 ± 0.007. We considered *Chrysomya megacephala*_FJ153270 and *Chrysomya rufifacies*_FJ839395 as misidentifications, and *Chrysomya rufifacies*_AY842670_AY842671 to be different from the other *Chrysomya rufifacies* individuals given the high sequence divergence (viz. mean p-distance = 0.10 ± 0.013). The mean p-distance between *Cochliomyia macellaria* and *Cochliomyia hominivorax* was 0.048 ± 0.009. The mean intra- and interspecific p-distances among all Chrysomyinae species (excluding *Phormia regina*) were 0.014 ± 0.014 and 0.046 ± 0.005, respectively (Table 3).

**Cyt *b***: The three EU and seven NA haplotypes of *Phormia regina* (from 17 specimens) formed two strongly supported clades (Figure 4) with a mean p-distance of 0.053 ± 0.009 between these two clades (Table 3). There were no cyt *b* sequences of *Phormia* in GenBank. Sequence divergence in *Phormia regina* within each continent was approximately a ten-fold lower, viz. EU: 0.002 ± 0.007 – NA: 0.005 ± 0.002 (Table 3). The mean p-distance between the three *Chrysomya* species was 0.046 ± 0.005. The mean intra- and interspecific p-distances among all Chrysomyinae species (excluding *Phormia regina*) were 0.003 ± 0.002 and 0.079 ± 0.007, respectively (Table 3).

**Supplementary figure 1.**
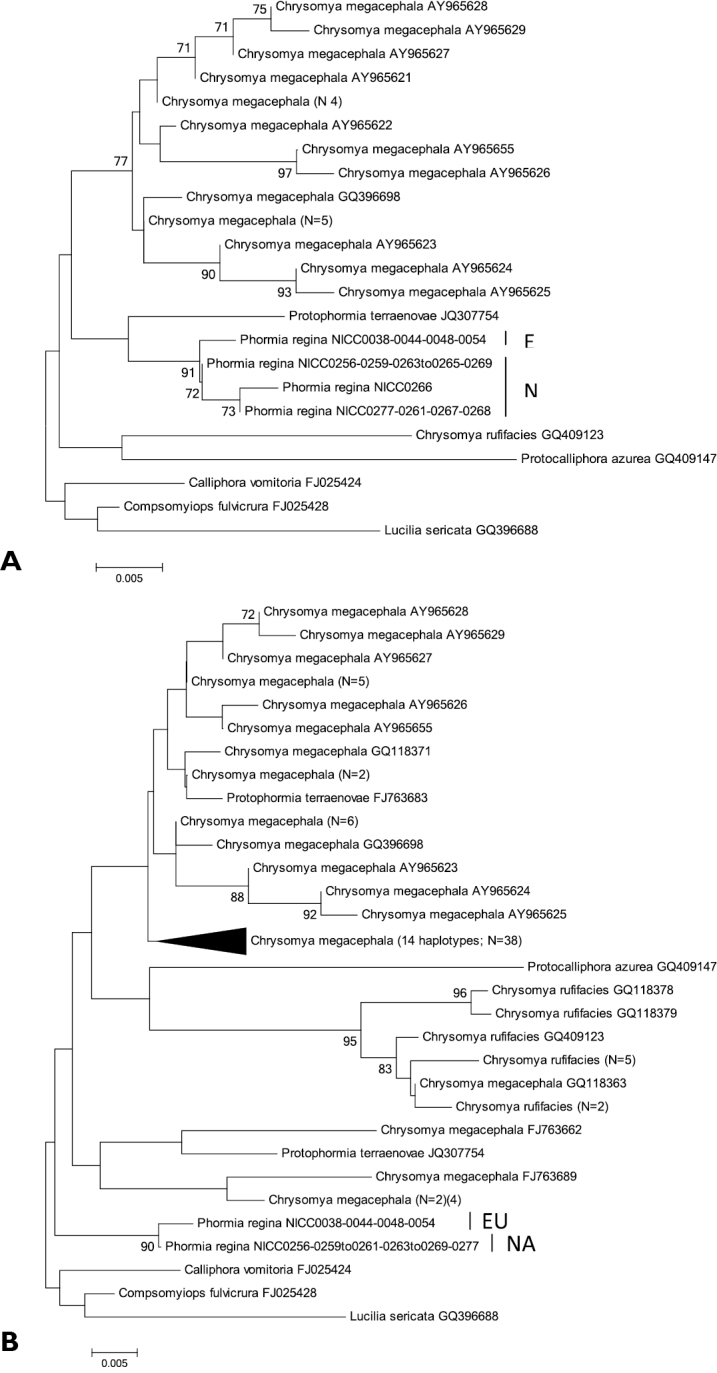
Neighbour-Joining tree (p-distances) of a 350 bp (**A**) and of a 251 bp (**B**) fragment of the mitochondrial 16S gene. Bootstrap values ≥ 70% are shown at the nodes. N gives the number of specimens of that haplotype. EU = *Phormia regina* haplotypes from W Europe; NA = *Phormia regina* haplotypes from N America.

**16S**: For the 350 bp dataset, the three NA 16S haplotypes (from 15 specimens) (mean within NA p-distance = 0.004 ± 0.003; Table 3) formed a well-supported clade, and formed a monophyletic group with the single EU haplotype (Supplementary figure 1A). The mean p-distance between the NA and EU haplotypes was 0.006 ± 0.003. The mean p-distance between *Chrysomya megacephala* and *Chrysomya rufifacies* was 0.040 ± 0.009. The mean intra- and interspecific p-distances among all Chrysomyinae species (excluding *Phormia regina*) were 0.014 and 0.023 ± 0.004.

For the 251 bp dataset, all eleven NA specimens had the same haplotype with a p-distance of 0.004 to the EU haplotype (four specimens) (Supplementary figure 1B). The mean p-distance between *Chrysomya megacephala* and *Chrysomya rufifacies* was 0.059 ± 0.012. The mean intra- and interspecific p-distances among all Chrysomyinae species (excluding *Phormia regina*) were 0.028 ± 0.009 and 0.038 ± 0.006, respectively (Table 3).

**Supplementary figure 2.**
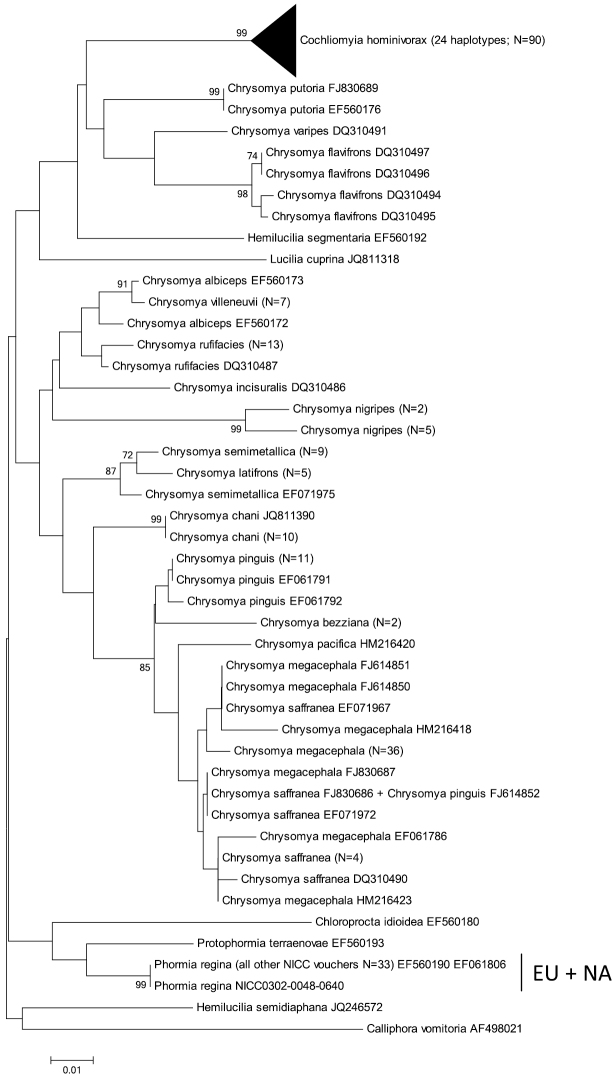
Neighbour-Joining tree (p-distances) of a 404 bp (229 bp without indels) fragment of the nuclear internal transcribed spacer 2 (ITS2). Bootstrap values ≥ 70% are shown at the nodes. N gives the number of specimens of that haplotype. EU = *Phormia regina* haplotypes from W Europe; NA = *Phormia regina* haplotypes from N America.

**ITS2**: Excluding indels, all *Phormia regina* specimens (36 specimens) had the same haplotype (Supplementary figure 2), except for *Phormia regina* NICC0302 that had a C instead of a T at position 219 of the alignment (p-distance = 0.003). *Phormia regina* NICC0640 had a deletion at position 201, and *Phormia regina* NICC0048 had an insertion of a G at position 270 of the alignment. Both specimens were from the same locality (Liège – Belgium) in W Europe. The p-distance between *Cochliomyia hominivorax* and *Cochliomyia macellaria* was 0.008 ± 0.001, that between *Hemilucilia segmentaria* and *Hemilucilia semidiaphana* was 0.106 ± 0.018, and the mean p-distance among 16 *Chrysomya* species was 0.085 ± 0.010. The mean intra- and interspecific p-distances among all Chrysomyinae species (excluding *Phormia regina*) were 0.008 ± 0.005 and 0.085 ± 0.011, respectively (Table 3).

**Supplementary figure 3.**
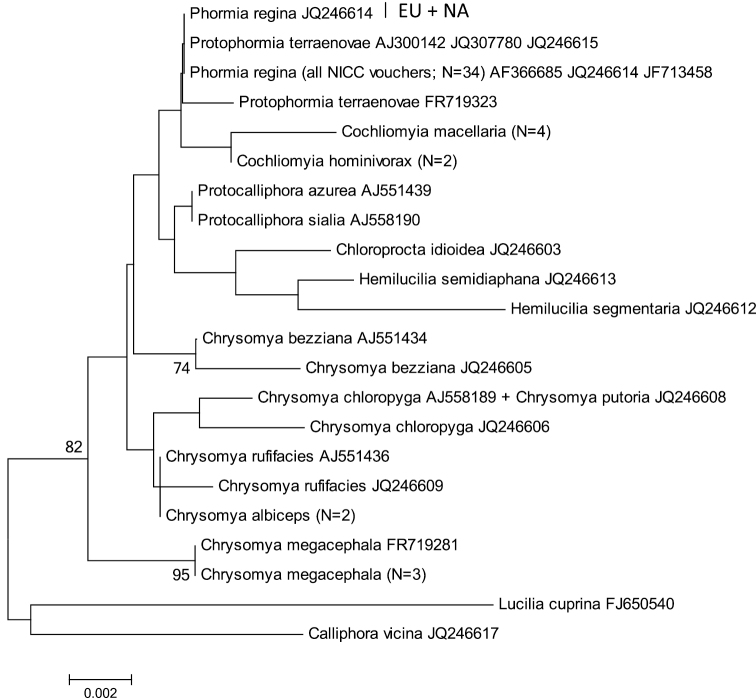
Neighbour-Joining tree (p-distances) of a 633 bp fragment of the nuclear 28S gene. Bootstrap values ≥ 70% are shown at the nodes. N gives the number of specimens of that haplotype. EU = *Phormia regina* haplotypes from W Europe; NA = *Phormia regina* haplotypes from N America.

**28S**: All 37 *Phormia regina* specimens had the same haplotype, except for *Phormia regina* JQ246614 from N America that had an AG insertion at positions 460-461 of the alignment (Supplementary figure 3). One haplotype of *Protophormia terraenovae* (three specimens with GenBank accession numbers AJ300142, JQ307780 and JQ246615) only differed by two indels from haplotype JQ246614 of *Phormia regina* (at positions 408 and 460-461) (the other *Protophormia terraenovae* haplotype differed at more positions). The mean p-distance between *Cochliomyia macellaria* and *Cochliomyia hominivorax* was 0.005, that between *Protocalliphora azurea* and *Protocalliphora sialia* was zero [an indel at position 439 (A) in *Protocalliphora azurea*) of the alignment], and that between *Hemilucilia semidiaphana* and *Hemilucilia segmentaria* was 0.013. The mean p-distance among the six *Chrysomya* species was 0.006 ± 0.002. The mean intra- and interspecific p-distances among all Chrysomyinae species (excluding *Phormia regina*) were 0.003 ± 0.004 and 0.007 ± 0.002, respectively (Table 3).

## Discussion

Desmyter and Gosselin (2009) and Boehme et al. (2012) found a mean sequence divergence of approximately 4% within a 304 bp and the barcoding COI region between N American and W European *Phormia regina*, respectively. We confirmed this COI divergence with newly sequenced material. Such a strong divergence at COI is common among insect species (e.g. Park et al. 2011a, b, Webb et al. 2012, Ng’endo et al. 2013). Moreover, we here show a similar degree of divergence at two other mtDNA genes, viz. COII (3.7%) and cyt *b* (5.3%). The ‘within-continent’ divergence in *Phormia regina* was very low (0.2-0.5% for the three genes) and comparable to the intraspecific differentiation of other Chrysomyinae (0.5% for COI, 1.4% for COII, 0.3% for cyt *b*). Hence, the high between-continent mtDNA differentiation, and low within-continent mtDNA divergence may hint at a taxonomic difference between the N American and W European populations. In order to evaluate this suggestion, we included all publicly available GenBank sequences from species of the subfamily Chrysomyinae for the four mtDNA and two nDNA gene fragments that we sequenced. The combined study of mtDNA and nDNA has proven valuable to disentangle the taxonomy of other calliphorid species (e.g. Nelson et al. 2007, Sonet et al. 2012).

On the one hand, our results show that the mean p-distance of other intrageneric interspecific comparisons (COI: 5–6.8%, COII: 4.8-5.9%, cyt *b*: 4.6%, 16S (251 bp): 5.9%), or among other Chrysomyinae species in general (COI: 6.6%, COII: 4.6%, cyt *b*: 7.9%, 16S (251 bp): 3.8%), are higher than the mean p-distances between N American and W European *Phormia regina* at the four mtDNA fragments (COI: 4%, COII: 3.7%, cyt *b*: 5.3%, 16S: 0.6%). For cyt *b* the NA-EU differentiation in *Phormia regina* is higher than that observed within other Chrysomyinae species (0.3%) yet still below the minimum interspecific p-distance (7.3%). On the other hand, for COI and COII, the NA-EU differentiation in *Phormia regina* is higher than the intraspecific differentiation in other Chrysomyinae species and well within the range of interspecific p-distances within Chrysomyinae. Yet, the low interspecific p-distance between some Chrysomyinae species may be due to misidentifications or may be the result of a natural process (e.g. hybridization, incomplete lineage sorting). Likewise, the high intraspecific variation within some species may be indicative of cryptic diversity (see further).

North American and W European *Phormia regina* were not differentiated at both nDNA fragments, and at the mtDNA 16S (< 1%), whereas interspecific p-distances in Chrysomyinae in general are substantial for ITS2 (8.5%) and 16S (3.8%). Moreover, the NA-EU differentiation in *Phormia regina* at these genes was even lower than the minimum intraspecific differentiation within other Chrysomyinae. This suggests that the variation at these genes in *Phormia regina* is intraspecific variation. Finally, we could neither detect color differences in 11 external characters, nor in the general shape of the male copulatory organs between N American and W European specimens. Evidently, a statistical analysis of more specimens (from a wider range of the species’ distribution) is necessary to reliably assess within and among population variation at these (and eventually other) morphological characters. For the time being, we consider the high differentiation at COI, COII and cyt *b*, but the low (16S, nDNA) or lack of (morphological) differentiation, as indicative of substantial intraspecific mtDNA sequence divergence, rather than as a species-level differentiation.

Our findings may have important implications for the use of *Phormia regina* in forensic and other scientific fields. Indeed, it has been suggested that the high variation in developmental times and growth curves of *Phormia regina* (e.g. Byrd and Allen 2001 and references therein) is partly due to differences in the population genetic structure (Picard and Wells 2009) and that therefore ecological data obtained from one population should not be generalized or extrapolated to other populations (Byrne et al. 1995). Interestingly, Marchenko (2001) reports a mean accumulated degree-days (from egg to adult) of 148 °C (lower development temperature: 11.4 °C) for Russian/Lithuanian *Phormia regina*, whereas a mean accumulated degree-days of 162 °C (lower development temperature: 11.16 °C) was found for N American *Phormia regina* (Yves Braet, unpublished preliminary results). Hence, the strong mtDNA divergence between N American and W European *Phormia regina* requires a sound comparison of the ecology of populations from both continents, especially since *Phormia regina* is a key species in the study of the physiology and neurology of insect feeding (e.g. Haselton et al. 2009, Larson and Stoffolano 2011, Ishida et al. 2012). Moreover, if locally diverged populations differ in their developmental biology, then this may affect the estimate of PMImin.

Intraspecific mtDNA divergence in other Chrysomyinae species is sometimes also high, viz. 4.3% for COI in *Chrysomya megacephala*, and 2.2%, 2.6% and 3.7% for COII in *Chrysomya megacephala*, *Chrysomya semimetallica* and *Chrysomya rufifacies*, respectively. Whereas these high intraspecific divergences may be due to hybridization/introgression or incomplete lineage sorting, they may also point to misidentifications. Obviously these issues are problematic if DNA barcoding of animals is only based on COI, as advocated by Hebert et al. (2003a, b). For instance, three *Chrysomya megacephala* specimens (KC135924, KC139925, KC135926) have a remarkably high p-distance of 8% with the other *Chrysomya megacephala* haplotypes and it would be advisable to re-identify these specimens. Also *Chrysomya semimetallica* shows much more intraspecific sequence variation (mean p-distance = 0.011 ± 0.003) as compared to other Chrysomyinae species but at the same time the species has a low mean interspecific p-distance with *Chrysomya albiceps* (p-distance = 0.017 ± 0.004).

Although there is no doubt that COI is a useful tool for the identification of forensically important Chrysomyinae species (Wells and Sperling 2001, Nelson et al. 2007, Wells and Williams 2007, Desmyter and Gosselin 2009, Boehme et al. 2012) not all species can be identified with COI. For instance, there is very low mean interspecific p-distance of 0.006 ± 0.002 between *Chrysomya megacephala* (excluding the three aforementioned haplotypes), *Chrysomya cabrerai*, *Chrysomya saffranea* and *Chrysomya pacifica* (the first two even share a haplotype) (see also Harvey et al. 2008). Therefore, other genes (or gene fragments) might help to overcome the shortcomings of the sole use of COI as molecular identification tool. We here showed that also COII may be a good DNA barcode marker in the Chrysomyinae. Indeed, the mean interspecific p-distance at COII is 4.6%, whereas the mean intraspecific distance is much lower (1.4%). Yet, the amount of Chrysomyinae COII data that is currently available in public libraries such as GenBank (194 sequences representing 108 haplotypes from 20 species), is rather limited compared to the amount of COI data (339 sequences representing 180 haplotypes from 36 species) (Table 1). Moreover, the problems inherent to misidentifications and introgression also apply to COII (or any other DNA marker). For instance, *Chrysomya megacephala* FJ153270 shares a haplotype within the *Chrysomya rufifacies* clade, and *Chrysomya rufifacies* FJ839395 shares a haplotype within the *Chrysomya megacephala* clade. Also other species share haplotypes such as *Chrysomya semimetallica* and *Chrysomya latifrons*. The other two mtDNA fragments (cyt *b* and 16S) cannot yet be evaluated as DNA barcode markers because of insufficient sequence data (cyt *b*: 32 sequences representing 20 haplotypes of five species; 16S: 39 sequences representing nine haplotypes of six species) (Table 1), but both have been shown to discriminate sufficiently between other dipteran species of forensic interest (Vincent et al. 2000, Li et al. 2010).

So far, the forensically important species within the Chrysomyinae belong to the genera *Chrysomya*, *Cochliomyia*, *Paralucilia*, *Protophormia* and *Phormia*. A number of COI reference datasets of these species are available (e.g. Wallman and Donnellan 2001, Wells and Sperling 2001, Nelson et al. 2007, Wells and Williams 2007, Harvey et al. 2008, Desmyter and Gosselin 2009, Boehme et al. 2012) and they seem to work well to identify most forensically important species. Yet, it is important to also include species without a clear forensic interest in (local) reference databases because this will improve the assessment of species boundaries which, in turn, may help to reach a stable taxonomy.

In conclusion, we observed substantial differentiation between N American and W European *Phormia regina* at the mtDNA genes COI, COII and cyt *b*, but not at the 16S rDNA and the nDNA genes ITS2 and 28S. Moreover, we neither detected any morphological differentiation between specimens from both continents. We therefore consider the strong mtDNA divergence between specimens from both continents as intraspecific variation. This differentiation has to be taken into account when using *Phormia regina* in forensic casework or physiological studies. Finally, the use of COII as a DNA barcode marker in the Chrysomyinae seems to perform as good as the standard COI barcode region.

## Appendix 1

**Supplementary table 1.** Sampling localities, voucher numbers and GenBank numbers of the *Phormia regina* and *Protophormia terraenovae* that were sequenced in this study.

Species	continent/country	country/state	city/county	latitude/longitude	voucher no.	GenBank accession no.					
						COI	COII	16S	cyt *b*	ITS2	28S
*Phormia regina*	Europe	Belgium	Andrimont	50°36'36"N, 5°54'36"E	NICC 0323	KJ908102					
			Andrimont	50°36'36"N, 5°54'36"E	NICC 0324	KF908103					
			Andrimont	50°36'36"N, 5°54'36"E	NICC 0325	KF908104					
			Andrimont	50°36'36"N, 5°54'36"E	NICC 0326	KF908105					
			Andrimont	50°36'36"N, 5°54'36"E	NICC 0327	KF908106					
			Andrimont	50°36'36"N, 5°54'36"E	NICC 0328	KF908107					
			Andrimont	50°36'36"N, 5°54'36"E	NICC 0329	KF908108					
			Andrimont	50°36'36"N, 5°54'36"E	NICC 0331	KF908109					
			Andrimont	50°36'36"N, 5°54'36"E	NICC 0332					KF908199	KF908234
			Andrimont	50°36'36"N, 5°54'36"E	NICC 0334					KF908200	KF908235
			Andrimont	50°36'36"N, 5°54'36"E	NICC 0336					KF908201	
			Auderghem	50°49'05"N, 4°24'41"E	NICC 0032	KF908069					
			Custine	49°41'41"N, 3°49'28"E	NICC 0314	KF908100					
			Genk	50°57'13"N, 5°29'56"E	NICC 0038			KF908054			
			Genk	50°57'13"N, 5°29'56"E	NICC 0355	KF908110					
			Hastière	50°13'14"N, 4°49'39"E	NICC 0027				KF908153	KF908171	
			Laeken	50°53'10"N, 4°22'36"E	NICC 0044		KF908126	KF908055	KF908154	KF908172	
			Liège	50°37'49"N, 5°33'17"E	NICC 0048		KF908127	KF908056	KF908155	KF908173	KF908205
			Liège	50°37'49"N, 5°33'17"E	NICC 0638						KF908202
			Liège	50°37'49"N, 5°33'17"E	NICC 0640				KF908169	KF908203	KF908236
			Liège	50°37'49"N, 5°33'17"E	NICC 0641						KF908237
			Messancy	49°35'31"N, 5°48'54"E	NICC 0317	KF908101					
			Schaerbeek	50°51'34"N, 4°22'25"E	NICC 0035	KF908070					
			Schoonaarde	51°00'17"N, 4°00'05"E	NICC 0359	KF908111					
			Schoonaarde	51°00'17"N, 4°00'05"E	NICC 0360	KF908112					
			Steendorp	51°07'25"N, 4°14'49"E	NICC 0054		KF908128	KF908057			KF908206
			Toernich	49°39'01"N, 5°45'07"E	NICC 0024					KF908170	KF908204
		France	Sarreguemines	49°06'50"N, 7°04'18"E	NICC 0295	KF908092					KF908227
			Sarreguemines	49°06'50"N, 7°04'18"E	NICC 0296	KF908093			KF908166	KF908195	KF908228
		Germany	Frankfurt	50°06'41"N, 8°40'49"E	NICC 0301	KF908094				KF908196	KF908229
			Frankfurt	50°06'41"N, 8°40'49"E	NICC 0302	KF908095				KF908197	KF908230
			Frankfurt	50°06'41"N, 8°40'49"E	NICC 0303	KF908096			KF908167		
			Frankfurt	50°06'41"N, 8°40'49"E	NICC 0304	KF908097					
			Frankfurt	50°06'41"N, 8°40'49"E	NICC 0305				KF908168		KF908231
			Frankfurt	50°06'41"N, 8°40'49"E	NICC 0306	KF908098				KF908198	KF908232
			Frankfurt	50°06'41"N, 8°40'49"E	NICC 0307	KF908099					KF908233
	USA	Indiana	Rensselaer Co.	40°56'12"N, 87°09'03"W	NICC 0275	KF908088	KF908150				KF908222
			Rensselaer Co.	40°56'12"N, 87°09'03"W	NICC 0276	KF908089	KF908151			KF908192	KF908223
			Rensselaer Co.	40°56'12"N, 87°09'03"W	NICC 0277	KF908090	KF908152	KF908068		KF908193	KF908224
			Rensselaer Co.	40°56'12"N, 87°09'03"W	NICC 0278					KF908194	KF908225
			Rensselaer Co.	40°56'12"N, 87°09'03"W	NICC 0279	KF908091					KF908226
		Texas	Brazos	32°39'41"N, 98°07'19"W	NICC 0265	KF908080	KF908140	KF908063			KF908212
			Brazos	32°39'41"N, 98°07'19"W	NICC 0266	KF908081	KF908141	KF908064	KF908161	KF908184	KF908213
			Brazos	32°39'41"N, 98°07'19"W	NICC 0267	KF908082	KF908142	KF908065	KF908162		KF908214
			Brazos	32°39'41"N, 98°07'19"W	NICC 0268		KF908143	KF908066	KF908163	KF908185	KF908215
			Brazos	32°39'41"N, 98°07'19"W	NICC 0269		KF908144	KF908067	KF908164	KF908186	KF908216
		Virginia	Pr. Williams Co.	38°31'20"N, 77°17'22"W	NICC 0260	KF908075	KF908135		KF908158	KF908179	
			Pr. Williams Co.	38°31'20"N, 77°17'22"W	NICC 0261	KF908076	KF908136	KF908060	KF908159	KF908180	KF908209
			Pr. Williams Co.	38°31'20"N, 77°17'22"W	NICC 0262	KF908077	KF908137		KF908160	KF908181	KF908210
			Pr. Williams Co.	38°31'20"N, 77°17'22"W	NICC 0263	KF908078	KF908138	KF908061		KF908182	
			Pr. Williams Co.	38°31'20"N, 77°17'22"W	NICC 0264	KF908079	KF908139	KF908062		KF908183	KF908211
		Washington	Snohomish Co.	47°54'46"N, 122°05'53"W	NICC 0270	KF908083	KF908145			KF908187	KF908217
			Snohomish Co.	47°54'46"N, 122°05'53"W	NICC 0271	KF908084	KF908146		KF908165	KF908188	KF908218
			Snohomish Co.	47°54'46"N, 122°05'53"W	NICC 0272	KF908085	KF908147			KF908189	KF908219
			Snohomish Co.	47°54'46"N, 122°05'53"W	NICC 0273	KF908086	KF908148			KF908190	KF908220
			Snohomish Co.	47°54'46"N, 122°05'53"W	NICC 0274	KF908087	KF908149			KF908191	KF908212
		Wyoming	Park Co.	44°31'52"N, 108°57'40"W	NICC 0255	KF908071	KF908130			KF908174	
			Park Co.	44°31'52"N, 108°57'40"W	NICC 0256		KF908131	KF908058		KF908175	
			Park Co.	44°31'52"N, 108°57'40"W	NICC 0257	KF908072	KF908132		KF908156	KF908176	
			Park Co.	44°31'52"N, 108°57'40"W	NICC 0258	KF908073	KF908133			KF908177	KF908207
			Park Co.	44°31'52"N, 108°57'40"W	NICC 0259	KF908074	KF908134	KF908059	KF908157	KF908178	KF908208
*Protophormia terraenovae*	Europe	Belgium	Andrimont	50°36'36''N, 5°54'36''E	NICC 0030	KF908113					
			Andrimont	50°36'36''N, 5°54'36''E	NICC 0095	KF908115					
			Andrimont	50°36'36''N, 5°54'36''E	NICC 0096	KF908116					
			Andrimont	50°36'36''N, 5°54'36''E	NICC 0336	KF908117					
			Andrimont	50°36'36''N, 5°54'36''E	NICC 0337	KF908118					
			Andrimont	50°36'36''N, 5°54'36''E	NICC 0338	KF908119					
			Andrimont	50°36'36''N, 5°54'36''E	NICC 0339	KF908120					
			Andrimont	50°36'36''N, 5°54'36''E	NICC 0340	KF908121					
			Andrimont	50°36'36''N, 5°54'36''E	NICC 0341	KF908122					
			Andrimont	50°36'36''N, 5°54'36''E	NICC 0342	KF908123					
			Auderghem	50°49'05''N, 4°24'41''E	NICC 0033	KF908114					
			Auderghem	50°49'05''N, 4°24'41''E	NICC 0358	KF908124					

## Appendix 2

Text file with the alignments for all the gene fragments studied. (doi: 10.3897/zookeys.365.6202.app2) File format: Text file (txt).

**Explanation note:** Text file with the alignments (fasta format) for all the gene fragments studied (COI, COII, cyt *b*, 16S (251 bp), 16S (350 bp), ITS2 and 28S, respectively).
